# First Metatarsophalangeal Joint Arthrodesis: A Narrative Review of Fixation Constructs and Their Evolution

**DOI:** 10.7759/cureus.14458

**Published:** 2021-04-13

**Authors:** Ketrick L LaCoste, Nicholas A Andrews, Jessyca Ray, Whitt M Harrelson, Ashish Shah

**Affiliations:** 1 Orthopaedic Surgery, University of Alabama at Birmingham, Birmingham, USA

**Keywords:** first metatarsophalangeal arthrodesis, fixation, fusion, construct, screw, plate, staple, orthopaedic surgery, foot and ankle

## Abstract

First metatarsophalangeal (MTP) joint arthrodesis is a surgical procedure in which the first metatarsal head is fused to the proximal phalanx of the great toe in order to permanently stiffen the first MTP joint. It was originally proposed as a treatment for severe cases of hallux valgus deformity, but the procedure’s indications and utilization have expanded since its initial development. Despite a wide variety of indications, first MTP arthrodesis has been shown to have reliable, satisfactory outcomes. As a result, the development of a wide array of surgical approaches, joint preparation techniques, and fixation devices used in the procedure has occurred. In this narrative review, we highlight the evolution of fixation constructs used in first MTP arthrodesis in order to provide a frame of reference for the various types of fixation constructs available.

## Introduction and background

First metatarsophalangeal (MTP) joint arthrodesis is a surgical procedure in which the distal aspect of the first metatarsal bone is fused to the proximal aspect of the proximal phalanx in order to permanently stiffen the first MTP joint in an effort to eliminate pain caused by arthritic degeneration of the first MTP joint or other deformities of the forefoot [1-4]. It was originally described by Clutton in 1894 as a treatment for severe cases of hallux valgus, but, over the years, its indications have since expanded [5]. It is now most commonly used as means for surgical treatment of end-stage arthritis of the first MTP joint [6], also known as hallux rigidus, as well as for a salvage operation in the case of previously failed cheilectomy, resection arthroplasty, implant arthroplasty, or severe hallux valgus deformity [7-10]. As a result of successful outcomes, first MTP arthrodesis has become a standard procedure in the treatment of disorders of the forefoot and first ray [11-16]. Various recent studies have reported fusion rates ranging from 88% to 100% with low revision rates and patient satisfaction scores ranging from 73% to 100% [1,17]. Over time, there has been an evolution in the types of implants used in first MTP arthrodesis as the procedure’s use and popularity have increased, from simple screw fixation to more recently developed plates with continuous compression. This narrative review provides a summary of first MTP arthrodesis fixation constructs of the past, present, and near-future.

## Review

Originally, Clutton proposed fixation with an ivory peg, but many other fixation devices have been developed since the initial proposal of first MTP arthrodesis. Many are still being used even as new devices and configurations are being developed and studied, adding to the potential options available to surgeons performing first MTP arthrodesis [5]. This evolving and ever-expanding list of fixation constructs has provided surgeons with many options when selecting hardware for first MTP arthrodesis, each with its own unique benefits and limitations. Current options for fixation devices include screws in various configurations, Kirschner wires (K-wires), Steinmann pins, monofilament wires, biodegradable rods, both locking and non-locking plates, memory compression staples, and various combinations of these fixation devices [7,11,18-21].

Screws

**Figure 1:**
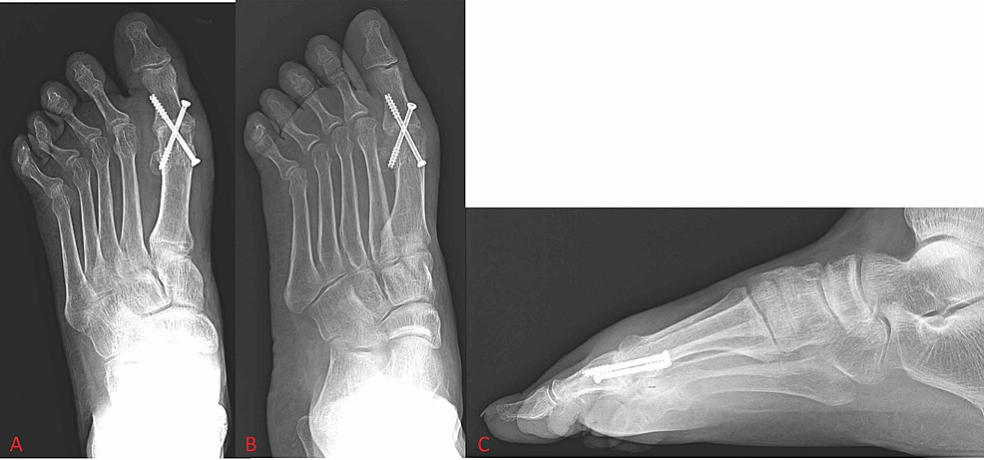
Foot X-ray shows crossed-screw fixation of the first metatarsophalangeal joint. Figure 1A and Figure 1B show anterior-posterior and oblique views of the foot, respectively. Figure 1C shows a lateral view of the foot.

Screws were the first fixation constructs to see regular use and are still being used today due to their versatility. One of the earliest detailed techniques for MTP arthrodesis, from McKeever in 1952, describes its fixation construct as a stainless-steel screw with a washer that was inserted through the proximal phalanx and seated into the metatarsal shaft [15]. Fixation with screws has since changed drastically, but screws are still common means of fixation in first MTP arthrodesis. Currently, screw fixation is primarily in the form of a supplementary lag screw [11,16,20] or as the fixation construct itself, most commonly as a single screw, two parallel screws, or two crossed screws (Figure 1) [7,16,22-24]. Studies have shown these methods of fixation to be acceptable options as they work well in patients with good bone quality and cause less postoperative irritation and discomfort due to their relatively low profile. The lower profile of screw fixation is also useful in scenarios in which joint space is limited, such as fixation in children or in cases where deformities prevent the use of plates. Crossed-screw fixation can provide an acceptable amount of biomechanical stability in these scenarios [7,22]. One limitation to the use of screws as a sole fixation construct is that patients must remain non-weight-bearing for a longer period of time than if another construct, such as a plate, or even a combination of constructs is used. More recent studies are beginning to investigate the use of new types of screws in new configurations in an effort to improve arthrodesis techniques with regard to invasiveness, postoperative complications, fusion rates, stability, and cost of each fixation device [7,22].

Kirschner wires (K-wires), Steinmann pins, sutures, and biodegradable rods

**Figure 2:**
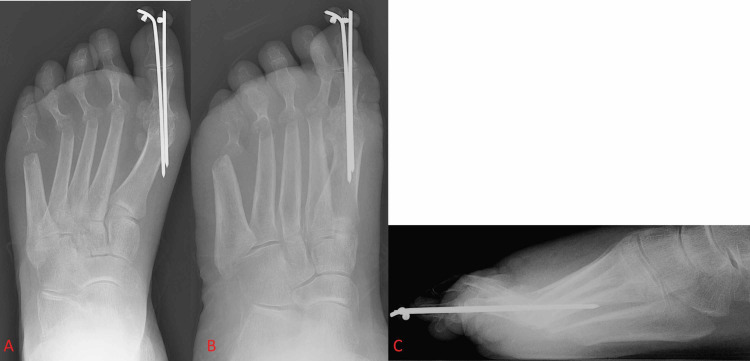
Foot X-ray shows Kirschner wire fixation of the first metatarsophalangeal joint. Figure 2A and Figure 2B show anterior-posterior and oblique views of the foot, respectively. Figure 2C shows a lateral view of the foot.

While screws and plates are generally the fixation constructs of choice for first MTP arthrodesis today, several others have been employed in the past. Several studies in the 1970s explored alternative methods of fixation with Kirschner wires plus cast immobilization and with tension sutures after reshaping of the bones within the first MTP joint [25-26]. Years later, Steinmann pins were investigated as a potentially viable means of fixation [27]. Currently, the use of K-wires and Steinmann pins in first MTP arthrodesis is primarily in patients with severe deformities due to rheumatoid arthritis or other seronegative arthritic conditions where traditional internal fixation methods are not possible (Figure 2) [27-28]. New variations on these techniques are being developed and utilized in combination with other fixation devices, but recent studies have shown that K-wires, suture techniques, and pins alone are generally inadequate means of long-term fixation [22]. Shortly after the boom in first MTP fusion in the mid-to-late 1980s, when operations were still largely being performed with Steinmann pins as a fixation construct, new fixation constructs began seeing use in first MTP arthrodesis. One of these unique constructs was a biodegradable poly-L-lactide rod that began to be utilized for patients with rheumatoid arthritis in the late 1980s and early 1990s, but their inconsistent outcomes, difficult implantation technique, and high cost prevented them from seeing extensive use [21-22].

Plates

**Figure 3:**
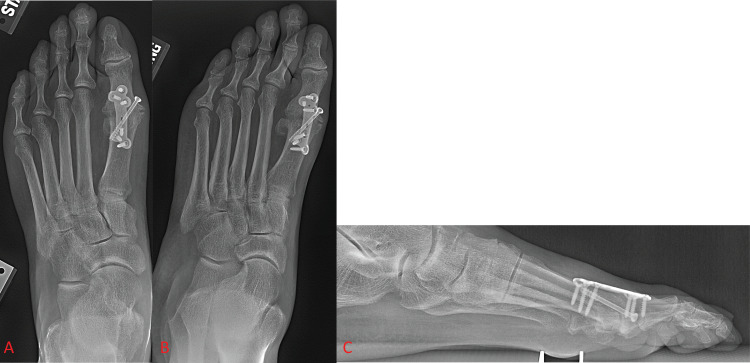
Foot X-ray shows dorsal locking plate fixation of the first metatarsophalangeal joint with supplementary lag screw fixation. Figure 3A and Figure 3B show anterior-posterior and oblique views of the foot, respectively. Figure 3C shows a lateral view of the foot.

Fixation and fusion with dorsal plating has been regularly employed in first MTP arthrodesis since the early 1990s. Plates have been shown to confer a biomechanically superior fusion construct compared to previously used methods of fixation by providing more compression and stiffness to the fused joint, which is crucial in preventing non-union [1,7,11,29]. Surgeons are also provided with a great deal of flexibility when choosing the exact variation of the plating method, allowing them to select their constructs on a case-by-case basis. When plates first began seeing use in first MTP arthrodesis, the original preferred method of plate fixation was a dorsal non-locking plate with a compressive lag screw (Figure 3) [20,30].

**Figure 4:**
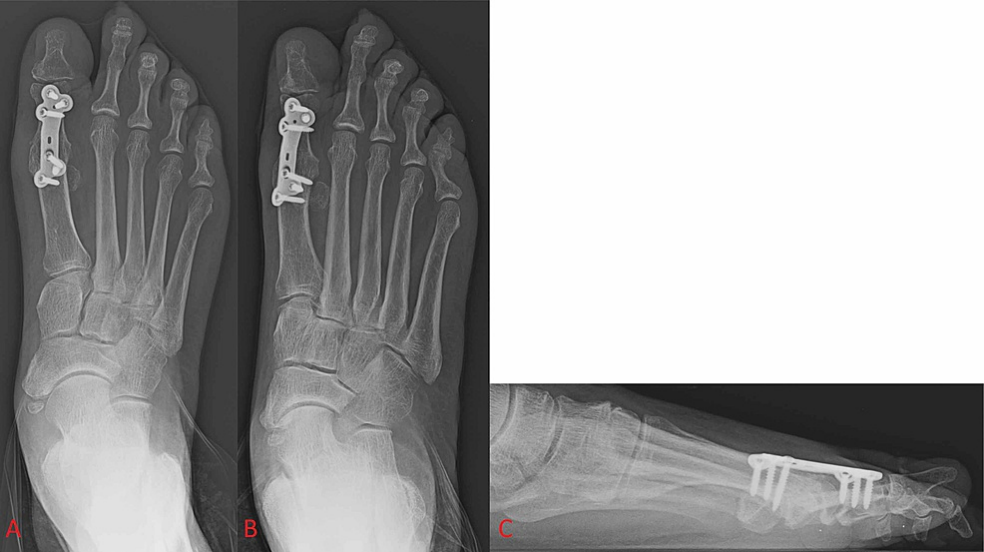
Foot X-ray shows dorsal locking plate fixation of the first metatarsophalangeal joint without a supplementary interfragmentary lag screw. Figure 4A and Figure 4B show anterior-posterior and oblique views of the foot, respectively. Figure 4C shows a lateral view of the foot.

Various studies have shown the biomechanical superiority of plates to other fixation devices such as Steinmann pins and variable pitch screws, though there is no significant difference in union rates between fixation with the different varieties of plates and screws [11,17,28,31]. After the success of plating techniques at large in the field of orthopaedics, locking plates were developed and began to rapidly increase in popularity in the early-to-mid 2000s due to their advantages of built-in compression, establishing rigidity, and minimizing screw loosening as well as causing less disruption of the underlying cortical bone (Figure 4). These advantages led dorsal locking plates with or without a compressive lag screw to be the new preferred plating technique in first MTP arthrodesis for many surgeons [32-36].

The popularity of dorsal plating has caused further advancements in the design of the plates themselves. Manufacturers now offer dorsal plates in various configurations including different degrees of dorsiflexion in order to provide a more anatomically favorable fusion of the first MTP joint. Since plates were adopted for use in first MTP joint arthrodesis, many studies have been conducted comparing locking plates, non-locking plates, lag screws, and their various combinations as fixation devices. Despite the potentially conflicting nuances among studies, all seem to favor the more recently developed locking compression plates due to their superior stability as a construct while maintaining comparable clinical and radiological outcomes when compared to the other, less stable constructs [11-12,16,26]. Plating techniques have also been shown to be superior to other fusion constructs in cases with reduced bone mineral density and poor bone quality [7,24,31]. However, plates are not without their disadvantages. They require a larger incision and lie on top of the bones that they fuse. A larger incision is more likely to produce more postoperative discomfort and can predispose to complications such as infection and delayed wound closure. Also, their dorsal positioning can cause a prominence of the hardware and, therefore, discomfort with activity in some patients. Additionally, the size of the construct eliminates it as a feasible option for fusion in the case of recently established techniques for minimally invasive arthrodesis of the first MTP joint [7,37]. Nevertheless, they are the current construct of choice in first metatarsophalangeal arthrodesis for many orthopaedic surgeons.

Novel constructs

**Figure 5:**
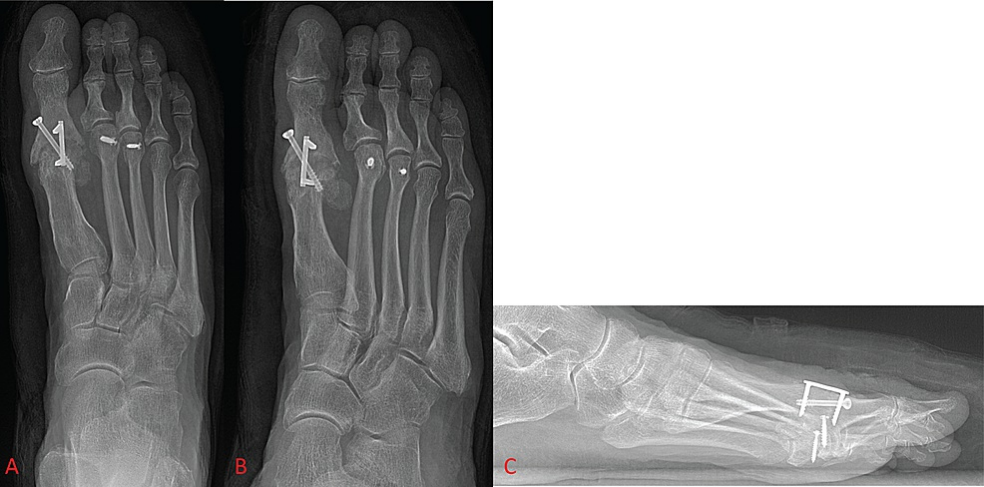
Foot X-ray shows memory compression staple fixation of the first metatarsophalangeal joint with supplementary interfragmentary lag screw fixation. Figure 5A and Figure 5B show anterior-posterior and oblique views of the foot, respectively. Figure 5C shows a lateral view of the foot.

It is also of note that more techniques and fixation devices are constantly being developed, adjusted, and combined. Recently, fully threaded, headless screws have been used in first MTP arthrodesis and have been found to be significantly stiffer than locking plates while maintaining similar values for plantar gapping and load-to-failure when the joint is prepared arthroscopically [7]. The memory compression staple is another more recently developed fixation construct that has also seen use in first MTP arthrodesis (Figure 5). They have also displayed similarly satisfactory clinical and radiological outcomes when compared to the most commonly employed fixation constructs in first MTP arthrodesis [29]. However, more investigation is needed as other studies reported data in favor of the “gold standard” plating techniques, which could be due in part to the heterogeneity of the studies themselves [7,26,29,38].

**Figure 6:**
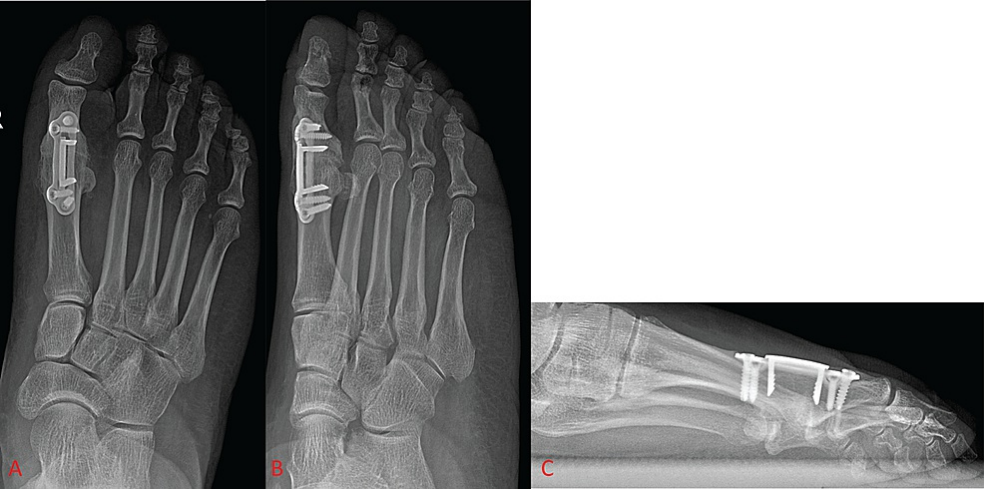
Foot X-ray shows hybrid dorsal locking plate with memory staple compression fixation of the first metatarsophalangeal joint. Figure 6A and Figure 6B show anterior-posterior and oblique views of the foot, respectively. Figure 6C shows a lateral view of the foot.

The next step in the evolution of fixation systems for the MTP joint will also include hybrid dorsal locking plates with staple compression and screw fixation of the plate. These hybrid systems allow for the creation of a pseudo-plantar tension band that provides continuous compression at both the plantar and dorsal aspects of the first MTP joint. The hybrid plates can be used both with and without interfragmentary screws, as the nitinol staples can provide sufficient compression across the joint surface alone even when not placed within a hybrid dorsal plate construct (Figure 6). This potentially offers a measurable decrease in operative time due to the simplicity of inserting a staple versus placement of an interfragmentary screw. Additionally, the continuous compression afforded by the use of nitinol staples is important, as it serves to help prevent the formation of gaps that can occur due to osteoclastic resorption or by other means when using constructs that utilize a static compression construct, such as an interfragmentary compression screw [39-40].

**Figure 7:**
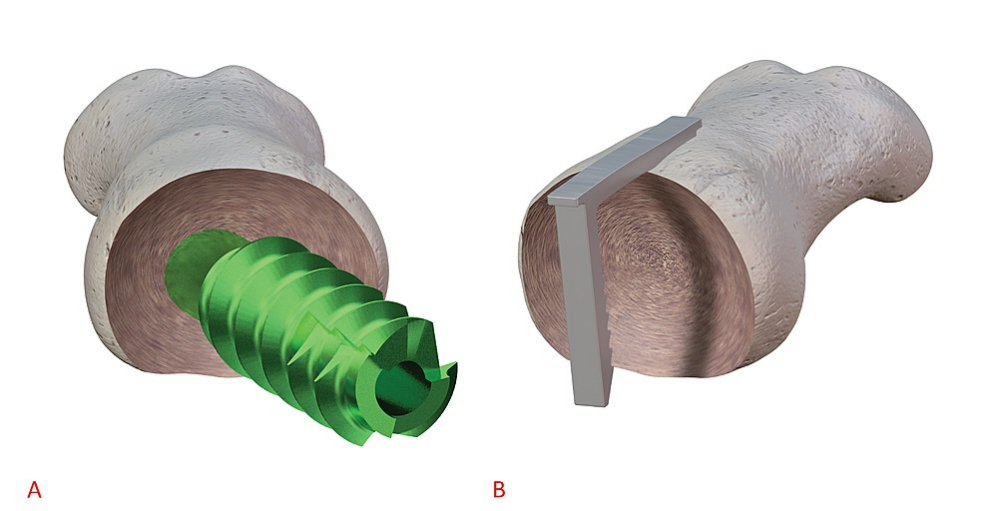
Three-dimensional model compares the amount of disruption of the cancellous bone surface between interfragmentary lag screws and memory compression staples in first metatarsophalangeal arthrodesis. Figure 7A shows a large amount of disruption caused by a lag screw passing directly through the first metatarsophalangeal joint. Figure 7B shows that memory compression staples can provide dynamic compression to the first metatarsophalangeal joint without any contact to the cancellous bone surface of the joint.

Notably, gapping is a known risk factor for non-union in foot and ankle arthrodesis [41]. This construct should also allow for maximal joint surface area contact due to the memory staple having no contact with the cancellous surfaces being fused while the dorsal plate provides the construct with stability (Figure 7). This is important as it is widely accepted that achieving maximal cancellous surface contact is a critical component for the formation of bony union. This novel combination has promising basic science rationale, and clinical trial data could prove beneficial in establishing this construct as another viable implant option in first metatarsophalangeal arthrodesis. More clinical studies will be needed to evaluate the efficacy of this new construct.

## Conclusions

Historically, the views on the best methods for first metatarsophalangeal arthrodesis have largely been under debate. The wide variety of options for both joint preparation techniques and selection of fixation constructs has kept this debate alive since the procedure’s inception. As new methods for fixation are developed and used alongside the older, more traditional ones, this debate will likely continue as more contemporary options are studied. Additionally, factors unrelated to the constructs and fusion techniques themselves, such as time to weight-bearing post-operatively or joint preparation technique, can influence the fusion rates and long-term outcomes of each of these constructs. The current lack of consensus among studies evaluating new combinations of fixation techniques and fixation devices practically guarantee that more combinations will continue to arise and be investigated. It is therefore important to remember the options that were previously, and are currently, available in order to select the best possible technique and implant for each patient. Surgeons should monitor the development of new techniques and implants as the field of orthopaedics expands.
